# Oral dirofilariasis mimicking endodontic failure: a case report

**DOI:** 10.1186/s12903-026-08115-x

**Published:** 2026-03-17

**Authors:** Aleksandra Karkle, Ksenija Silina, Ilze Akota, Angelika Krumina, Anete Vaskevica, Katrina Gardovska, Laura Neimane, Anda Slaidina

**Affiliations:** 1 Department of Conservative Dentistry and Oral Health, Rīga Stradiņš University, Riga, LV-1007 Latvia; 2 Department of Oral and Maxillofacial Surgery and Oral Medicine, Rīga Stradiņš University, Riga, LV-1007 Latvia; 3 Department of Prosthetic Dentistry, Rīga Stradiņš University, Riga, LV-1007 Latvia; 4 Rīga Stradiņš University Institute of Stomatology, Riga, LV-1007 Latvia; 5 Department of Infectiology, Rīga Stradiņš University, Riga, LV-1007 Latvia; 6 Department of Orthodontics, Rīga Stradiņš University, Riga, LV-1007 Latvia

**Keywords:** Acute apical abscess, Apical periodontitis, CBCT, Dirofilariasis, Parasitic Infection, Periapical lesion, Case report

## Abstract

**Background:**

Oral dirofilariasis is a rare parasitic infection that can mimic common dental pathologies. This report presents a unique case where dirofilariasis was mistaken for an acute apical abscess, highlighting diagnostic challenges and the importance of interdisciplinary collaboration.

**Case presentation:**

A 47-year-old male presented with a persistent, painless swelling in the maxillary anterior region (tooth 11). Initial history and radiographic examinations suggested an acute apical abscess following inadequate root canal treatment. However, during surgical exploration, a live nematode was discovered in the periapical lesion. The parasite was extracted, and histopathological analysis confirmed *Dirofilaria* spp. The patient underwent endodontic retreatment and achieved full recovery.

**Conclusions:**

This case underscores the need for clinicians to consider parasitic infections in atypical presentations of periapical pathology. Advanced imaging, thorough surgical assessment, and histopathological confirmation are essential in distinguishing rare infections from common dental diseases.

## Introduction

Dirofilariasis is a mosquito-borne zoonotic disease caused by nematodes of the *Dirofilaria* genus, primarily affecting dogs and wild canines, with humans usually serving as accidental hosts. Although humans are typically considered dead-end hosts, there have been documented reports of *microfilariae* detected in peripheral blood smears [1, 2]. The infection spreads through mosquito bites carrying third-stage larvae, which mature in both mosquito vectors and vertebrate hosts [3, 4]. While *Dirofilaria repens* typically form subcutaneous nodules, *Dirofilaria immitis* affects the pulmonary arteries and heart, causing severe complications in animals [3]. Though rare, human cases are increasing, highlighting its emerging zoonotic significance [5]. Infection in humans are often present as a solitary, slowly growing subcutaneous nodule, sometimes with swelling and pain, and may be asymptomatic, frequently leading to misdiagnosis as neoplastic or inflammatory disease [6, 7]. Asymptomatic cases pose diagnostic challenges and sustain transmission risk [8].

Recent studies indicate a northward spread of *Dirofilaria* spp., placing Estonia, Latvia, and Lithuania at risk [9, 10]. Climate change and animal movement through international sale contribute to this expansion, with *Dirofilaria repens* spreading more rapidly than *Dirofilaria immitis* [7, 10].

The parasite’s distribution is influenced by environmental factors allowing larval maturation rather than vector availability [11]. The northernmost confirmed location of an established *D. repens* life cycle in Europe is Tartumaa County, Estonia [12]. Finland documented its first imported dog case in 2014 and its first local human case in 2015 [11]. Poland confirmed its first autochthonous human infection in 2010, while cases have also been reported in other parts of Eastern Europe [13].

Addressing dirofilariasis requires a multidisciplinary approach. Studies in the Nordic-Baltic region emphasize the need for improved knowledge and preparedness [14, 15]. Consequently, clinicians practicing in both endemic and emerging areas must maintain awareness of atypical parasitic infections when evaluating persistent or unusual oral lesions [16].

Intraoral dirofilariasis remains exceptionally rare and, to date, has been reported in soft tissues of the oral cavity—most commonly the buccal mucosa, lips, and floor of the mouth—with no documented involvement of hard tissue structures such as alveolar bone or teeth [17–21]. However, no previous cases document *Dirofilaria* infection within a periapical bone lesion following failed endodontic treatment, making this presentation unique. Such rarity underscores the parasite’s atypical osseous extension beyond typical soft-tissue sites like buccal mucosa, prompting expanded differential diagnoses for persistent periapical pathologies.

## The clinical case

A 47-year-old male presented to the Oral Surgery Emergency Unit at Riga Stradiņš university Institute of Stomatology, Latvia, with a persistent swelling in the maxillary anterior region (tooth d11) that had been present for several weeks. The patient reported an unusual sensation but experienced no pain, fever, or systemic symptoms.

**Fig. 1:**
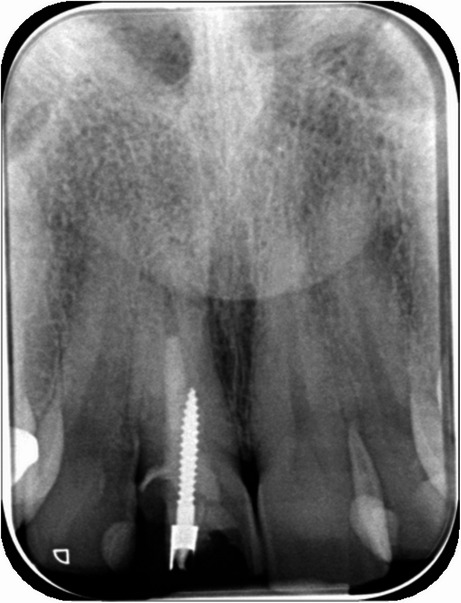
Pre-operative periapical X-ray

Clinical examination revealed a swelling in the buccal region of tooth d11. A positive palpation test was noted buccally, while the percussion test produced a negative result. Additionally, the periodontal probing depth was less than 3 mm in all six points around the tooth, and there was no mobility of the tooth. Radiographic evaluation, including a periapical X-ray (Fig. 1), showed inadequate root canal filling and a periapical radiolucency, indicative of periapical pathology. Following clinical assessment and radiological findings, the most common diagnosis initially established by the treating endodontist was an acute apical abscess.

**Fig. 2:**
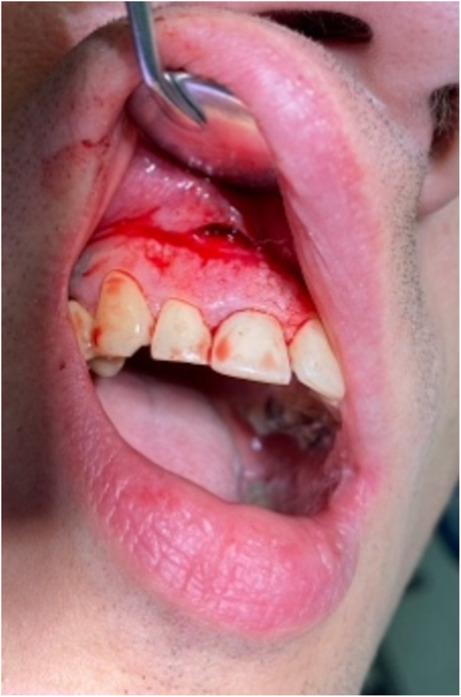
Surgical incision in progress revealing the unexpected presence of a parasite. The image captures the moment during the incision when the surgical team noticed movement within the lesion

**Fig. 3:**
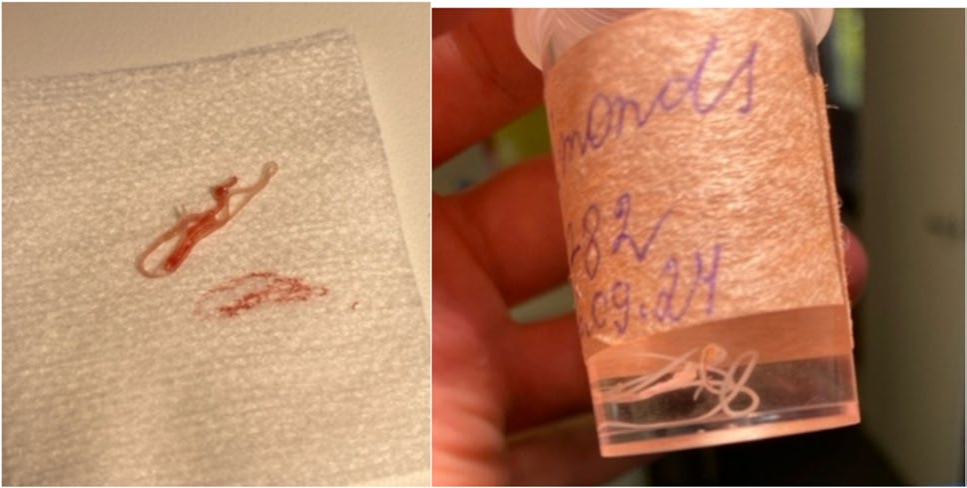
Extracted parasite

Based on the clinical and radiographic findings, an odontogenic aetiology was suspected, and a surgical procedure was planned on the same day for abscess drainage. For surgical intervention, local anesthesia with adrenaline 1:100 000 1,7 ml and an incision were made to drain the abscess. During the procedure, magnification revealed unexpected movement within the lesion (Fig. 2). A live nematode was extracted from the periapical bone lesion, followed by thorough curettage and chemical decontamination (Fig. 3). The wound was closed with a pressure bandage, and antibiotic therapy was prescribed due to the initial clinical suspicion of acute odontogenic infection. The extracted worm was sent for histopathological examination, which confirmed *Dirofilaria* spp.

**Fig. 4:**
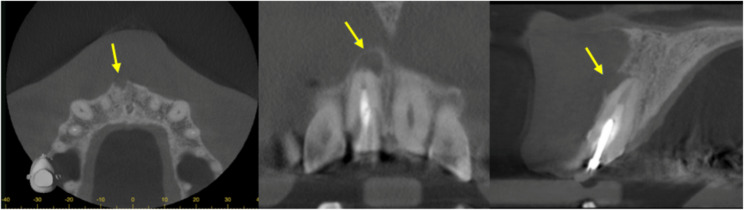
View of periapical defect in 3 axes. The yellow arrow indicates the area of bone destruction associated with the periapical lesion resulting from failed root canal treatment. This site is also where the parasite was detected

**Fig. 5:**
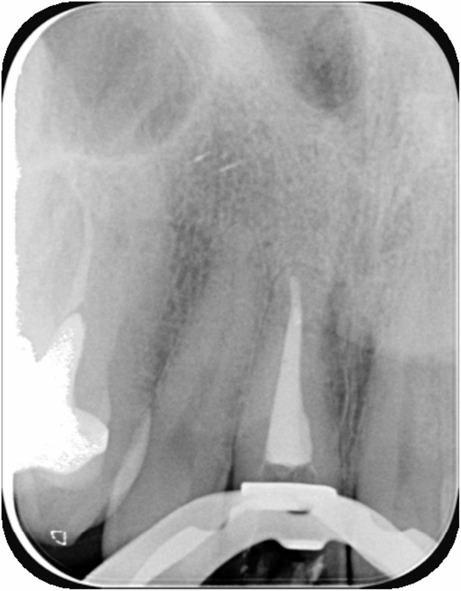
Post operative periapical X-Ray showing the root canal obturation

The patient was referred to an endodontist for further management 5 days later. After completing a questionnaire, the patient denied any recent travel abroad but stated that he owns a dog. Cone-beam computed tomography (CBCT) scan confirmed periapical bone loss, consistent with apical periodontitis (Fig. 4). Endodontic retreatment was performed in two sessions. During the first appointment, the old root canal filling material was removed, and the canal was mechanically shaped using rotary files. Chemomechanical decontamination was carried out with sodium hypochlorite 3% and ethylenediaminetetraacetic acid 17%. Root canal was filled with Calcium hydroxide as intracanal medicament and temporary filling was placed. At the second appointment, ultrasonic activation was used for enhanced irrigation, followed by canal obturation with bioceramic sealer and gutta-percha. A periapical radiograph confirms successful root canal obturation (Fig. 5). The canal was then temporarily sealed.

The procedure was performed under microscope magnification and aseptic conditions to eliminate residual infection and preserve the tooth. Following successful endodontic treatment, the patient was referred to a prosthodontist for further rehabilitation.

**Fig. 6:**
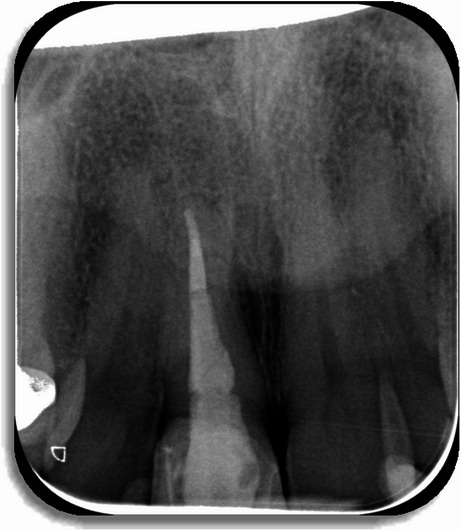
Six-month follow-up PA radiograph indicating apical area healing

**Fig. 7:**
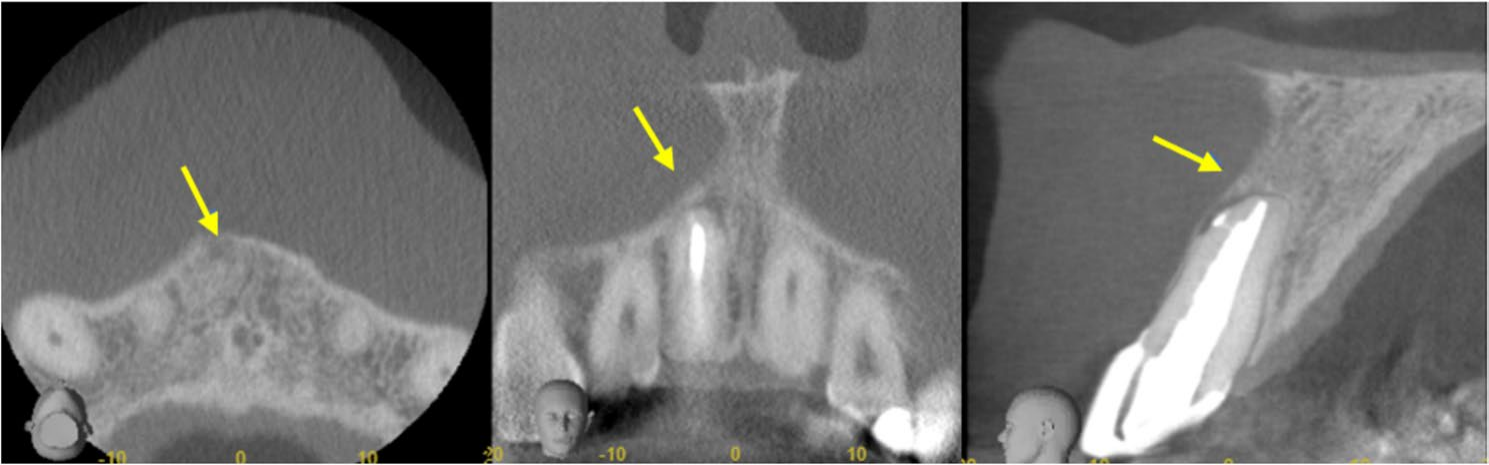
Three-axis CBCT view of the periapical region at 6-month follow-up. The yellow arrow indicates the previously affected area, now showing signs of bone regeneration and periapical healing following endodontic treatment and surgical intervention

The patient returned for a follow-up visit six months after the completion of treatment, during which a control radiograph confirmed complete healing (Fig. 6). One month later the patient also received a full-coverage crown (Fig. 7).

## Discussion and conclusions

### Discussion

In this case report, we present a rare instance of *Dirofilaria* infection identified within a periapical lesion that clinically and radiographically mimicked an endodontic abscess in a tooth with previous root canal treatment.

Clinical history revealed a tooth with previous root canal treatment and radiographic evidence of a periapical lesion. The patient presented with diffuse, nonspecific facial swelling accompanied by intraoral manifestations, which initially posed diagnostic challenges. The buccal swelling in the anterior maxillary region was characterized by periodic changes and an unusual sensation, leading to a preliminary clinical assessment in which dirofilariasis was not initially suspected, given the rarity of such presentations. Periapical radiography and CBCT demonstrated bone destruction consistent with a periapical lesion typically associated with odontogenic infection. The definitive diagnosis was established following surgical intervention and subsequent histopathological confirmation of an adult dirofilarial worm within a swollen gingival nodule. Clinically and radiographically, the lesion mimicked an odontogenic infection, resulting in an initial diagnosis of acute apical abscess.

Based on current evidence, *Dirofilaria *spp. are not considered a direct etiological factor in the development of apical periodontitis; therefore, the parasite most likely represented an incidental finding within an inflamed periapical region.

The pathophysiology of oral dirofilariasis involves accidental transmission, resulting in aberrant migration and localization of the parasite within oral tissues rather than reflecting a distinct or alternative transmission route [7, 22].

This case highlights the diagnostic challenges that such atypical presentations may pose for clinicians. Given the nonspecific clinical and radiographic features, differentiating parasitic infections from more common odontogenic pathologies remains challenging. Persistent periapical radiolucency may represent cysts, granulomas, foreign body reactions, or non-odontogenic lesions [23]. Soft-tissue parasitic infections, including dirofilariasis, should therefore be considered in cases of unexplained or persistent periapical radiolucency, particularly when asymptomatic swelling is present, radiographic features are atypical, or there is a lack of response to conventional diagnostic tests and endodontic treatment [22]. Clinicians should maintain a broad differential diagnosis, especially in endemic regions or when clinical features are unusual [20, 22, 24].

The gold standard for the diagnosis of dirofilariasis is the demonstration of an adult worm in tissue sections, characterized by a thick laminated cuticle with external longitudinal ridges and well-developed musculature [25, 26]. *Dirofilaria repens* can be identified by characteristic morphological features, including a defined pattern of ridges along the body circumference, and the presence of chord nuclei in cross-sections [27]. In the present case, despite the presence of granulomatous tissue reactions surrounding the lesion, the morphological features of the adult worm were sufficiently preserved to allow accurate identification. This preservation is particularly important, as inflammatory responses may obscure typical parasitic structures and complicate species differentiation, which is often relevant for epidemiological and geographic considerations [26]. While histopathological examination remains the diagnostic gold standard, molecular diagnostic techniques such as polymerase chain reaction (PCR) play a crucial complementary role in species identification, especially in cases where the parasite is fragmented or morphologically degraded [28, 29]. Despite inflammatory alterations associated with *Dirofilaria* infections, our findings illustrate the diagnostic value of thorough histopathological evaluation in revealing unusual parasitic infections presenting in association with odontogenic and periapical pathology.

*Dirofilaria* species, particularly *Dirofilaria repens*, are natural parasites of canines, typically residing in subcutaneous tissues, with human infection occurring via mosquito transmission of infective larvae [3]. Human dirofilariasis most commonly presents as subcutaneous nodules—often around the eyes, eyelids, and conjunctiva [4, 6, 26]. However, the occurrence of infection in the intraoral region, specifically within a periapical lesion, is notably uncommon. Previously reported intraoral dirofilariasis cases predominantly involved soft tissues [6, 18–20, 22, 24]. Case series and individual reports consistently describe presentation as asymptomatic, less commonly symptomatic submucosal or mucosal nodules, and there is no evidence of direct involvement of osseous or dental tissues [18, 20–22, 30, 31]. The present case uniquely demonstrates intraosseous periapical involvement, expanding the clinical spectrum.

Ultimately, human dirofilariasis is typically regarded as a single-worm infection. Consequently, excisional biopsy of the affected tissue is generally considered curative and is usually sufficient without the need for additional antiparasitic treatment. Preventive measures primarily focus on chemoprophylaxis in domestic animals and vector control, including mosquito bite prevention, particularly in endemic regions or during travel to such areas [4, 15]. This finding emphasizes not only the necessity of comprehensive assessments in dental practice but also the critical importance of recognizing parasitic infections in differential diagnoses for oral and facial swellings, especially in cases with prior failed treatments.

This case underscores the importance of heightened clinical awareness in endemic areas and highlights the need to consider rare non-odontogenic conditions in the differential diagnosis of apical pathology.

### Conclusion

In conclusion, our case of *Dirofilaria* infection presenting within an apical lesion in a previously treated tooth highlights the need for heightened clinical awareness of parasitic infections manifesting as atypical oral pathology. Although rare, oral dirofilariasis should be considered in the differential diagnosis of persistent or non-resolving periapical lesions, particularly in endemic or emerging regions. Early recognition, together with surgical exploration and histopathological confirmation, is essential for accurate diagnosis and effective management. A multidisciplinary approach involving endodontists, oral surgeons, and infectious disease specialists may be beneficial when addressing such unusual presentations. This case underscores the importance of continued vigilance and contributes to the growing body of evidence on zoonotic infections relevant to dental practice.

## Take-home messages

- Oral dirofilariasis can mimic common odontogenic pathology.
- Persistent periapical lesions require thorough evaluation.
- Surgical exploration and histopathology are critical.
- Awareness is essential in endemic and emerging regions.

## Data Availability

Data sharing is not applicable to this article as no datasets were generated or analysed during the current study.
